# Repeated vaccination against matched H3N2 influenza virus gives less protection than single vaccination in ferrets

**DOI:** 10.1038/s41541-019-0123-7

**Published:** 2019-07-09

**Authors:** Nedzad Music, Wen-Pin Tzeng, F. Liaini Gross, Min Z. Levine, Xiyan Xu, Wun-Ju Shieh, Terrence M. Tumpey, Jacqueline M. Katz, Ian A. York

**Affiliations:** 1 Influenza Division, National Center for Immunization and Respiratory Diseases, Centers for Disease Control and Prevention, 1600 Clifton Road, Atlanta, GA 30329 USA; 2 Infectious Diseases Pathology Branch, Division of High Consequence Pathogens and Pathology, National Center for Emerging and Zoonotic Infectious Diseases, Centers for Disease Control and Prevention, Atlanta, GA USA; 3 Present Address: Seqirus, A CSL Company, 50 Hampshire Street, Cambridge, MA 02139 USA

**Keywords:** Adaptive immunity, Vaccines, Influenza virus

## Abstract

Epidemiological studies suggest that humans who receive repeated annual immunization with influenza vaccine are less well protected against influenza than those who receive vaccine in the current season only. To better understand potential mechanisms underlying these observations, we vaccinated influenza-naive ferrets either twice, 10 months apart (repeated vaccination group; RV), or once (current season only group; CS), using a prime-boost regimen, and then challenged the ferrets with A/Hong Kong/4801/2014(H3N2). Ferrets that received either vaccine regimen were protected against influenza disease and infection relative to naive unvaccinated ferrets, but the RV group shed more virus, especially at the peak of virus shedding 2 days post infection (*p* < 0.001) and regained weight more slowly (*p* < 0.05) than those in the CS group. Qualitative, rather than quantitative, differences in the antibody response may affect protection after repeated influenza vaccination.

## Influenza: Repeat vaccination impairs protection

Epidemiological evidence suggests that influenza vaccination in prior years can reduce the efficacy of influenza vaccination compared to vaccine given in the current season alone. Ian A. York and colleagues of the Centers for Disease Control and Prevention, USA investigate this phenomenon using the human relevant ferret model of influenza infection. Ferrets undergoing repeat vaccination — twice 10 months apart — show quantitatively similar antibody and cell-mediated immune responses as those given a single current-season vaccination. However, qualitatively the responses elicited by the current season vaccine alone are superior — with animals showing less dramatic loss of body weight, milder fever, reduced virus shedding and quicker recovery of lymphocyte numbers. Although less protective than current-season vaccination alone, repeated vaccination offers better protection than mock vaccination.

## Introduction

Influenza A viruses are common human respiratory pathogens that infect hundreds of millions of people and cause 290,000–646,000 deaths globally per year.^1,2^ In addition to these seasonal epidemics, novel influenza viruses that have not previously circulated among humans occasionally cross from animal reservoirs, leading to global pandemics in the naive population.^3^ Annual influenza vaccination is the primary prevention strategy against seasonal influenza. In many countries, annual influenza vaccination is recommended only for high-risk individuals, whereas in the United States it is recommended for people 6 months of age and older without contraindications.^4^ Annual vaccination has been justified because influenza viruses constantly undergo antigenic drift, requiring periodic vaccine updates, and because vaccine-induced antibody titers decline relatively rapidly.^5^

The effectiveness of influenza vaccines can vary between subtypes and from year to year. The reasons for low vaccine effectiveness (VE) are complex and may include host factors such as age, health and immune status, as well as poor antigenic matches due to virus drift^6,7^ or egg-adaptive changes,^8–10^ and poor immunogenicity.^11^ In 2017–2018, overall VE for the A(H1N1)pdm09 component of influenza vaccine was about 64%, whereas for the A(H3N2) subtype VE was about 24%.^12^

A growing list of studies have suggested that receipt of influenza vaccine in prior years may diminish the effectiveness of the current-season’s vaccinations. The possibility that repeated influenza vaccination might reduce protective immunity, was first raised several decades ago,^13^ but subsequent studies were inconclusive or found that repeated vaccination was effective.^14–19^ However, a number of recent studies^20–26^ and some^27–29^ but not all^30^ meta-analyses have also concluded that repeated vaccination may be associated with reduced VE.

Several explanations as to why receiving influenza vaccination in 1 year might reduce its effectiveness in the following year have been proposed.^28^ The simplest possibility is that the effect is an artifact of study design, and reflects uncorrected confounders.^31^ Another possibility is that, when the virus strains in the initial and the repeated vaccine are antigenically matched but the circulating virus strain is drifted, the immune response may be too focused on the vaccine strains and less effective against the challenge virus (“negative interference”, or the “antigenic distance hypothesis”).^16,28^ Conversely, when the virus strains in the initial and the repeated vaccine are mismatched, the immune response to the features conserved between each may become amplified, increasing the components of the response that are less effective against the most recent viruses (“original antigenic sin hypothesis”).^28^ A fourth possibility is antibody sequestration, in which the antibodies induced by the initial immunization bind to subsequent vaccine antigens and prevent their exposure to the immune system.^32,33^ Finally, the “infection block” hypothesis suggests that by preventing highly immunogenic influenza infections, vaccination prevents individuals from gaining this mode of priming and protection before the repeated vaccination.^13,34^ Of course, more than one mechanism may lead to a reduced response to repeated vaccination, whether simultaneously or in different influenza seasons.

The ferret is considered to be the most relevant small-animal model for influenza infection.^35,36^ Human influenza strains infect ferrets without prior adaptation, and induce disease symptoms similar to those of humans. Although the ferret model is not as well characterized as the mouse model, a number of reagents and techniques for evaluating ferret immune responses and influenza pathogenesis have been recently developed.^37–42^ Using ferrets enabled us to test the effect of repeated versus single vaccination on protection against influenza with fewer confounders than in humans with complex immune histories due to prior vaccination and/or infection, as well as directly testing protective efficacy by challenge. Since influenza vaccine efficacy against the A(H3N2) viruses has been relatively low in recent years,^43,44^ we focused on this component of the vaccine for challenge studies. We found that ferrets receiving influenza vaccine in the current season only were more protected against challenge than were those receiving vaccine in sequential seasons, although the latter were better protected than were non-immunized ferrets.

## Results

### Repeated vaccination results in similar or higher serological responses as current-season vaccination

**Fig. 1:**
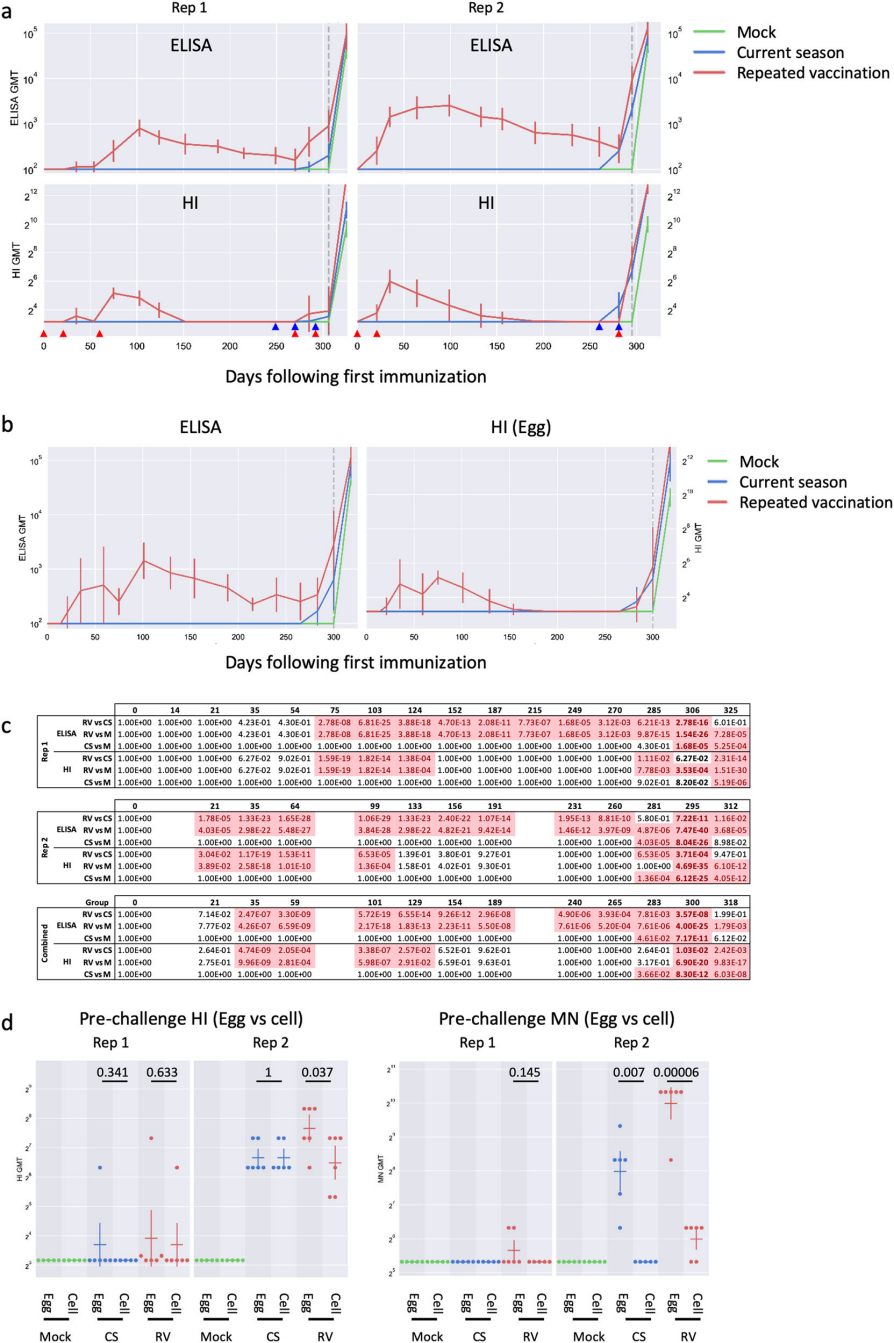
Serological responses to HK/4801. **a** ELISA (left panels) and HI (right panels) assays were performed using egg-grown HK/4801 on blood samples collected from ferrets undergoing various immunization regimens. Timing of immunizations is indicated at the bottom of the charts. Red arrows: repeat vaccination immunizations; blue arrows: current-season immunizations. Animals not receiving vaccine at a time point were mock immunized with PBS injections. The time of challenge with wild-type HK/4801 is indicated with a vertical gray dashed line. Left panels: replicate 1; right panels: replicate 2. Error bars represent one standard deviation; six ferrets per group. **b** As above, with replicates combined. **c** Statistical significances of the differences between groups, as measured using a linear mixed model with repeated measures. *P-*values < 0.05 are highlighted. **d** Blood samples were collected 4 days before viral, and the serological response to egg-adapted HK/4801 (as included in the vaccine) or to cell-grown HK/4801 (the challenge virus) was measured using HI assays (left two panels), and MN assays (right two panels). Dots represent individual ferrets; horizontal bars represent geometric mean titers (GMT); error bars represent one standard deviation. *P*-values (two-sided Student’s *t* test) are shown on the charts

These experiments were performed in two independent replicates using ferrets from two different suppliers, and using commercial quadrivalent inactivated influenza vaccine (QIV) from two different manufacturers but containing identical virus strains. Ferrets in the two experiments responded somewhat differently to immunization, although the overall picture was similar. All ferrets responded to immunization as measured by ELISA (Fig. 1a, upper panels), although two ferrets in the CS group, in Rep 1, had ELISA titers that were at the limit of detection (titer of 100). Ferrets in the first replicate (Rep 1) achieved HI geometric mean titers (GMT) of 35.6 (RV) or 15.1 (CS) after an initial prime and two booster vaccinations (timing of immunizations is indicated with arrows on Fig. 1a). Ferrets in the second replicate (Rep 2) achieved GMT against egg-grown HK/4801 of 63.5 (RV) or 100.8 (CS) after a single boost. In both groups, the RV GMTs were significantly higher than those of the CS groups at the time of challenge (Fig. 1a–c; day 306 (Rep 1), 295 (Rep 2), or 300 (combined)).

Ferrets were challenged with wild-type HK/4801 that was grown on cells, preventing development of the egg-adaptive mutations that occur in the high-yield vaccine viruses (Supplementary Fig. 1). These egg-adaptive mutations lead to a degree of antigenic mismatch between the vaccine and challenge viruses, with a minor to moderate mismatch detectable as measured by HI (Fig. 1d, left panels) and a more marked mismatch when measured by microneutralization (MN) assays (Fig. 1d, right panels).

Serological responses to the A(H1N1)pdm09 component of the quadrivalent vaccines roughly paralleled those to HK/4801 (Supplementary Fig. 3). Following challenge, as expected, anti-HK/4801 titers increased to very high levels (Fig. 1a, b), while the serological response to A/California/07/2009(A(H1N1)pdm09) increased by ELISA but dropped by HI (Supplementary Fig. 3). This is presumably since ELISA measures all antibodies that bind to HA, including those to both the highly variable HA1 region and to the much more conserved HA2 region, while HI assays measure antibodies that bind to the HA1 region that is not conserved between H3N2 and H1N1 influenza viruses.

### Repeated vaccination leads to less protection against influenza symptoms

**Fig. 2:**
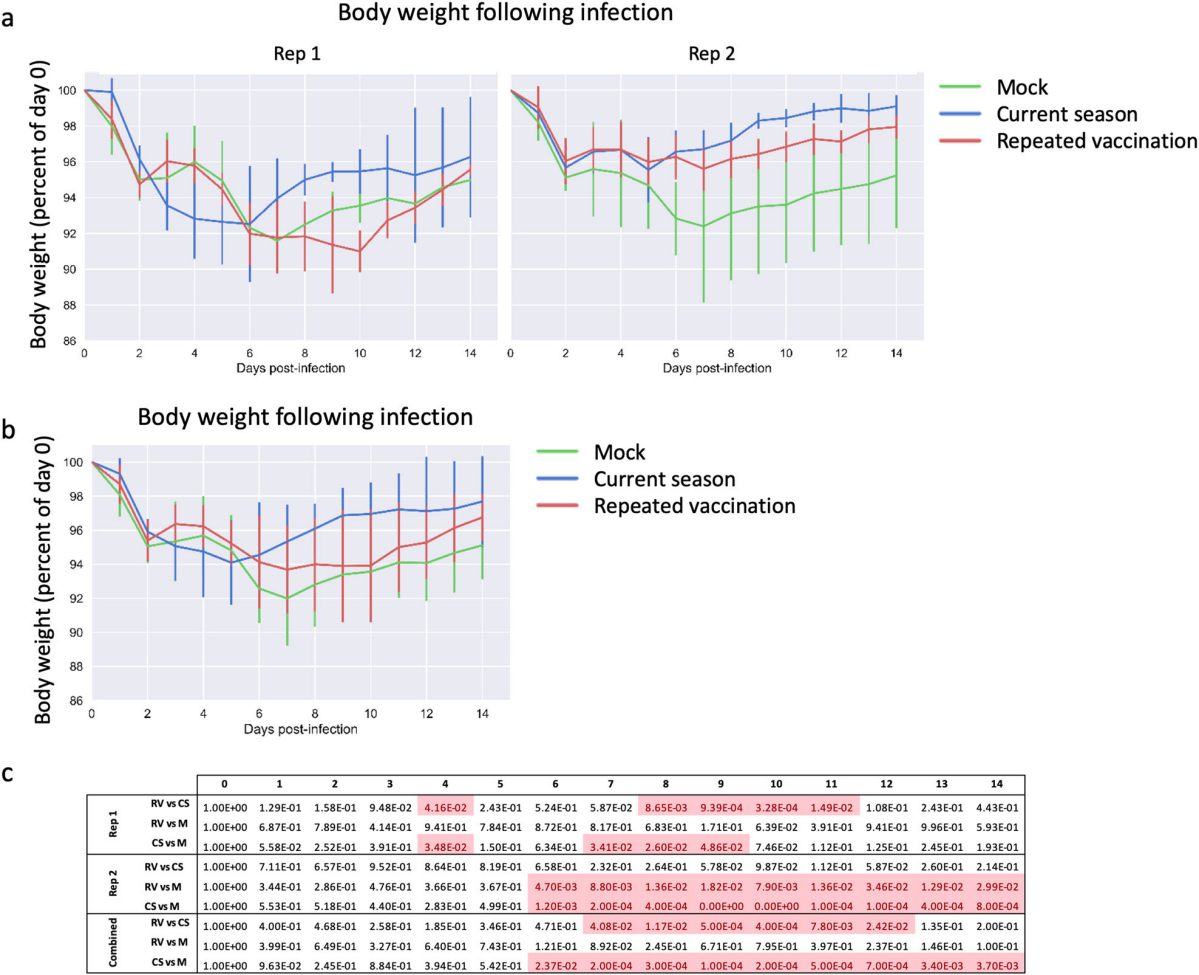
Changes in body weight following influenza challenge. **a** Ferrets were infected intranasally with cell-grown HK/4801, and body weight was measured daily for 2 weeks. Mean body weight of each group is shown for Rep 1 and 2, normalized to weight on the day of infection (day 0). Error bars represent one standard deviation; six ferrets per group until day 2 then three ferrets per group. **b** As above, with replicates combined. **c** Statistical significances of the differences between groups, as measured using a linear mixed model with repeated measures. *P-*values < 0.05 are highlighted

**Fig. 3:**
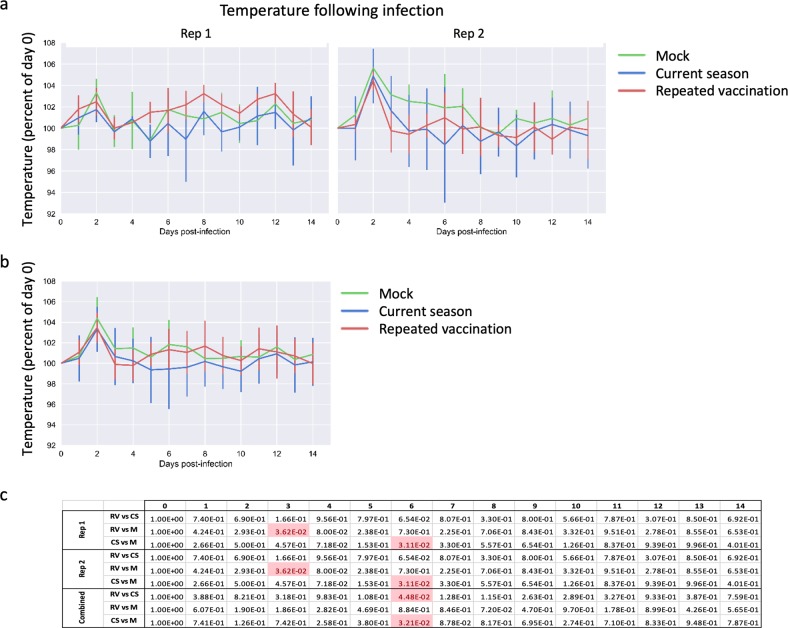
Changes in body temperature following influenza challenge. **a** Ferrets were infected intranasally with cell-grown HK/4801, and body temperature was measured daily for 2 weeks. Mean temperature per group is shown for Rep 1 and Rep 2, normalized to temperature on the day of infection (day 0). Error bars represent one standard deviation; six ferrets per group until day 2 then three ferrets per group. **b** As above, with replicates combined. **c** Statistical significances of the differences between groups, as measured using a linear mixed model with repeated measures

Naive ferrets challenged with wild-type, cell-grown HK/4801 developed moderate disease. They lost an average of 8% of their body weight (relative to body weight on the day of challenge), with maximum weight loss on day 7 followed by gradual recovery; even 14 days post challenge, these ferrets had lost on average about 5% of their body weight (Fig. 2). They also developed the typical biphasic fever associated with influenza,^45^ peaking on day 2, and recurring on day 6 post challenge (Fig. 3). The ferrets that received QIV in the current season only (CS group) began to recover body weight after day 5, and by 14 days post challenge had recovered to nearly 98% of their starting body weight (Fig. 2a, b). By contrast, the ferrets that received vaccination in both the previous and the current season (RV group) showed significantly more weight loss than the CS group (Fig. 2b), with the difference being statistically significant (*p* < 0.05) on days 7 through 12 (Fig. 2c). This effect was significant in Rep 1, and when weights were combined, but was not statistically significant (*p* > 0.05) in Rep 2.

### Repeated vaccination leads to less reduction in virus shedding

Both RV and CS immunization reduced virus shedding in nasal washes post challenge (Fig. 3). However, the CS immunization was more effective than RV, since CS reduced the peak titers of virus shedding about 90% compared with mock-immunized ferrets, while peak shedding was unaffected by the RV immunization (Fig. 3a–c, day 3). After day 2, the CS and RV groups both shed lower virus titers and cleared virus earlier than did mock-vaccinated ferrets (Fig. 3a–c).

### Cell-mediated immunity is similar following repeated vaccination and current season vaccination

Peripheral blood samples were taken before challenge and the response of CD3+/CD4+ and CD3+/CD8+ T cells to in vitro stimulation with HK/4801 or CA/07 was analyzed. As expected from immunization with an inactivated vaccine, cell-mediated immune (CMI) responses were low and mainly consisted of a CD4+ T-cell response, with many ferrets showing no detectable CMI in either CD8+ or CD4+ T cells (Supplementary Fig. 4). Differences between the Mock, CS, and RV groups were not significant (*p* > 0.05).

### Histopathological changes are similar following infection after repeat vaccination and current-season vaccination

Lungs from three ferrets per group were taken on 2 days post challenge, when viral titers were highest, and analyzed by histopathology. In general, consistent with the clinical signs, the lungs exhibited only mild-to-moderate interstitial and perivascular inflammatory infiltrates composed mainly of lymphocytes and scattered macrophages (Supplementary Fig. 5a). Histopathological scores were low, averaging 0.6, 1.5, and 1.2 for the mock, CS, and RV groups, respectively (Supplementary Fig. 5b), and did not differ significantly.

### T-lymphocyte counts are higher following infection after current-season vaccination than repeat vaccination

The fractions of various leukocyte subsets in ferret peripheral blood were measured by flow cytometry on days 0–7, 9, 11, and 13 post challenge and normalized to each ferret’s day 0 counts. As previously described,^39,40^ T-cell subsets in unvaccinated ferrets rapidly dropped following infection (Fig. 5a, b), and then gradually recovered in a biphasic manner to the original levels or higher. Granulocytes (CD11b-positive cells) followed an opposite pattern. T-cell subsets in the RV and CS groups were similar to each other, but differed from the mock infection group, for approximately the first week following infection. During the second week (e.g., days 9 and 11 post infection), higher lymphocyte counts were observed in the CS group than the RV group (Fig. 5a–c).

## Discussion

**Fig. 4:**
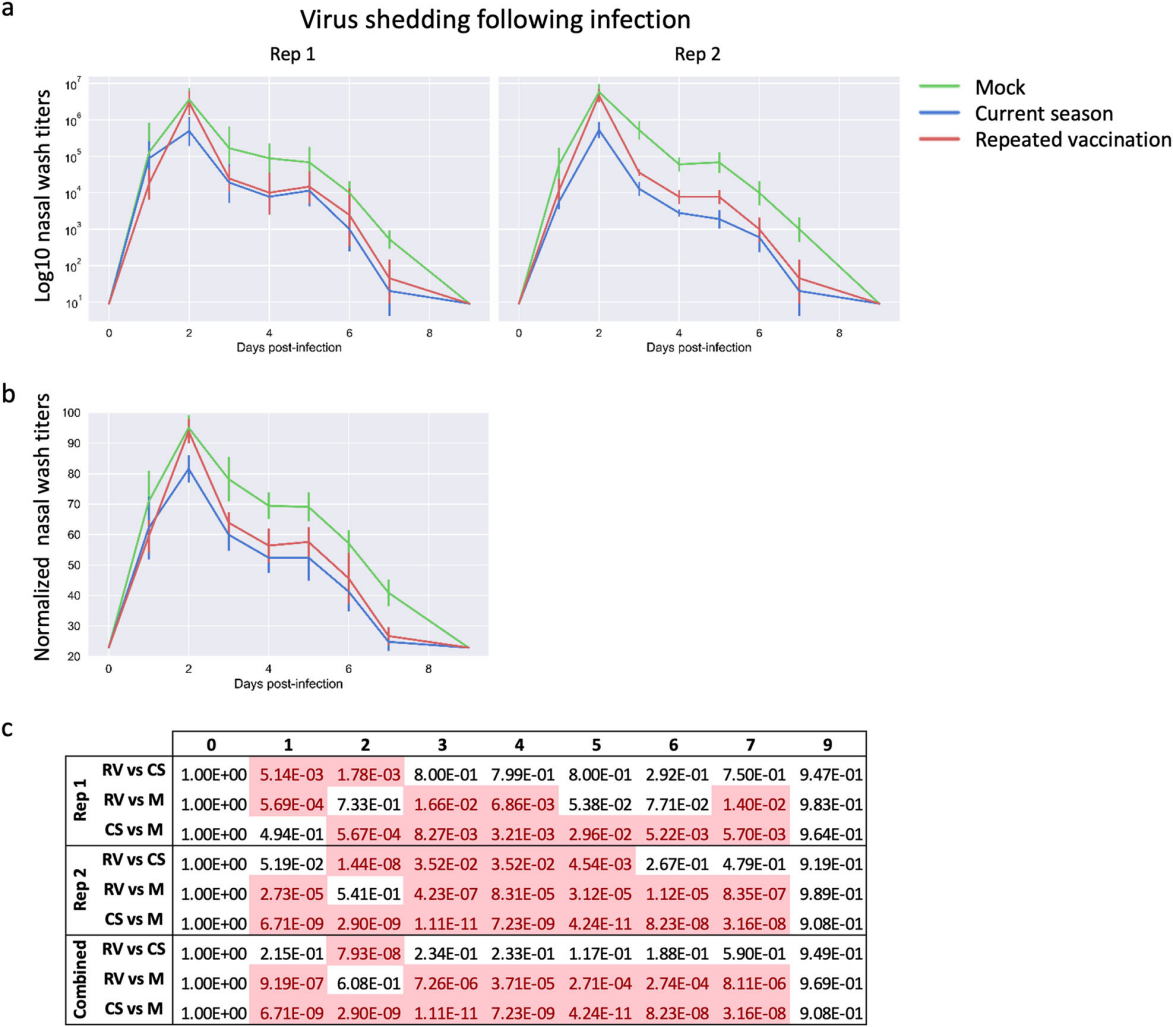
Virus shedding following influenza challenge. **a** Ferrets were infected intranasally with cell-grown HK/4801, nasal washes were taken on days 1–7 and 9, and virus titers were determined by TCID_50_. Titers are shown for Rep 1 and Rep 2. Error bars represent one standard deviation; six ferrets per group until day 2 then three ferrets per group. **b** As above, with replicates combined and normalized to the highest titer found in each replicate. **c** Statistical significances of the differences between groups, as measured using a linear mixed model with repeated measures

Epidemiological data suggest that humans who receive influenza vaccinations in two or more consecutive seasons may be less well protected than those who receive vaccination in the current season only.^13,20–28^ Here, we demonstrate that a similar effect holds true using well-defined viral challenges in the ferret model, which is considered to be the most useful small-animal model for human influenza infection. Ferrets that were immunized with commercial QIV twice about 10 months apart showed more weight loss and slower recovery of body weight (Fig. 2), and shed higher titers of virus (Fig. 4) than those immunized only in the current season. Although virus shedding was most different between the RV and CS groups on day 2 post challenge, by several other measures, including weight loss, temperature, and PBL counts, ferrets in the RV and CS groups responded similarly to infection for the first 5–6 days, after which the CS animals showed more complete recovery than did the RV or mock-vaccinated animals.

**Fig. 5:**
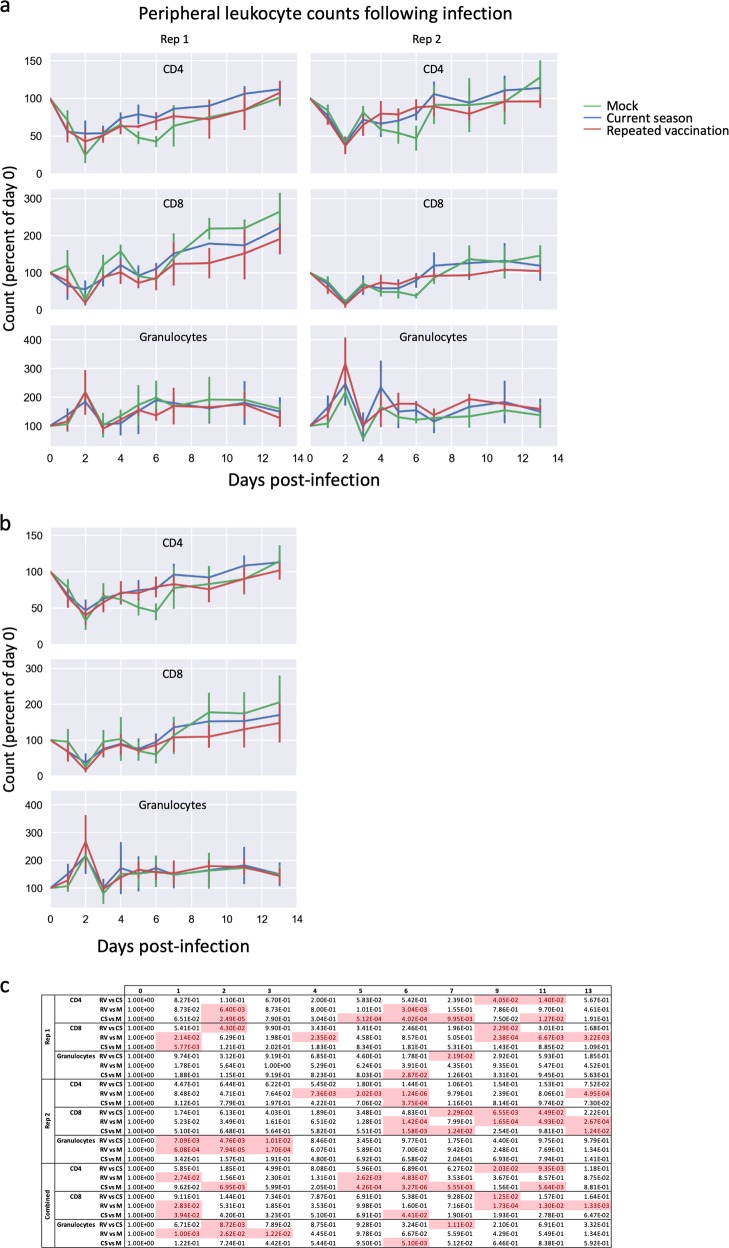
Changes in peripheral blood leukocyte counts following influenza challenge. **a** On days 1–7, 9, 11, and 13 following intranasal challenge with cell-grown HK/4801, blood samples were drawn and peripheral blood leukocytes were analyzed using flow cytometry as described in the Methods section. Mean percent of PBLs comprising CD4+ T cells (“CD4”), CD8+ T cells (“CD8”), or CD11b+ granulocytes (“granulocytes”) are shown as the mean of each group, with values normalized to values on the day of infection (day 0). Error bars represent one standard deviation; six ferrets per group until day 2 then three ferrets per group. **b** As above, with replicates combined. **c** Statistical significances of the differences between groups, as measured using a linear mixed model with repeated measures

Importantly, the RV group on average showed less weight loss (Fig. 2) and shed lower amounts of virus (Fig. 3) than did the unvaccinated ferrets, suggesting that while repeat vaccination may be suboptimal, it is still better than no vaccination at all. This is consistent with observations in humans.^21,24^

Various mechanisms have been proposed to explain the repeat vaccination phenomenon in humans. Some of these are unlikely to have played a part in these ferret experiments. Since ferrets were matched for age and sex and were housed in the same environments, uncorrected confounders^31^ are unlikely to have caused the differences. The ferrets were not exposed to influenza infection between the initial and repeated vaccination, ruling out the “infection block” hypothesis.^13,34,46^ Ferrets were all serologically naive to influenza at before immunization, and the A(H3N2) component of the initial and repeated vaccination was the same, since the WHO recommendations for this component did not change between the 2016/2017 and 2017/2018 Northern Hemisphere influenza seasons;^47,48^ therefore the “original antigenic sin hypothesis”, which in the context of the repeat vaccination phenomenon refers to differences in vaccine components,^28^ should not apply. Antigen sequestration by pre-existing antibodies^32,33^ seems unlikely, since the anti-influenza titers in the RV ferrets were very low by the time of the revaccination (Fig. 1).

Ferrets were immunized with a high-yield reassortant vaccine strain of HK/4801 that was adapted to eggs. Growth in eggs of contemporary A(H3N2) viruses typically leads to adaptive changes in the HA, including L194P and T160K, that alter the antigenicity of these viruses.^9,10^ The ferrets were challenged with HK/4801 that was propagated exclusively on MDCK-SIAT cells that do not drive adaptive changes in the HA, and the presence of the wild-type sequence L194 and T160 was confirmed by sequencing (Supplementary Fig. 1). HI and MN assays confirmed that the challenge virus was antigenically moderately different from the vaccine strain (Fig. 1d). Accordingly, it is possible that “negative interference” (the “antigenic distance hypothesis”^16,28^) may have occurred, in which the RV group focused their antibody response on the egg-adapted vaccine strain, leading to a low response to the cell-grown challenge strain. However, while the HI titers and, especially, the MN titers of the RV were indeed higher against egg-grown HK/4801 than to cell-grown HK/4801, the RV titers against cell-grown HK/4801 were still equal to or higher than those of the CS group (Fig. 1d). This observation differs from studies in humans that have found that repeat vaccination recipients tended to have lower B-cell responses and antibody titers than those immunized in the current season only.^24,33^ This difference may reflect the fact that, while the ferrets in this study were initially naive to influenza antigen, most adult humans have been repeatedly exposed to influenza (either through vaccination or through exposure to virus, or both).

Taken together, these data suggest that while RV induced an immune response that (at least measured by conventional assays such as ELISA, HI, MN, and cell-mediated responses) was quantitatively the same as or better than that induced by CS vaccination, the response was qualitatively different in ways that are not easily detected by standard influenza serological assays. Notably, the ratio of ELISA to HI antibody was much higher in the RV group (51.7 or 83.6 for the RV group for egg- and cell-grown HK/4801, respectively; 18.6 or 21.1 for the CS group for egg- and cell-grown HK/4801, respectively) (Fig. 1). This ratio has been used to estimate the amount of “non-neutralizing antibody” present,^49,50^ although the ability of neutralizing and non-neutralizing anti-influenza antibodies to actually protect against virus infection in vivo is poorly understood.^51,52^ It is possible that repeated vaccination with inactivated influenza vaccine induces a population of antibodies that are suboptimal for protection. Further research is needed to better understand the specific subpopulations of antibodies induced by various influenza vaccination regimens, and how the different subpopulations interact with virus infections. The ferret model should be useful in resolving many of these issues.

## Methods

### Experimental overview

Briefly, a prime-boost vaccine series, with commercial quadrivalent inactivated influenza vaccines containing the egg-adapted high-yield reassortant virus A/Hong Kong/4801/2014 (HK/4801) X-263B(H3N2) (“HK/4801”) as the A(H3N2) component, was administered to one group of six ferrets (“Repeated Vaccination”: RV). A second group of six ferrets group (“Current Season only”: CS) received mock immunization (phosphate-buffered saline (PBS) injection) at this time. Approximately 10 months later, both the RV and CS groups received the subsequent year’s inactivated egg-based vaccine, also containing HK/4801, and were subsequently challenged with HK/4801 that was propagated exclusively in cell culture rather than eggs. Since HK/4801 propagated in eggs rapidly accumulates egg-adaptive mutations (especially L194P and T160K) that alter its antigenic profile,^9,10^ the challenge virus was therefore not perfectly antigenically matched to the vaccine. A third group of ferrets were mock vaccinated (injected with phosphate-buffered saline (PBS) at both time periods. (In one replicate, during the 10-month period during which ferrets were housed, one of the “Mock vaccinated” group became moribund and was euthanized due to disseminated idiopathic myofasciitis of ferrets, unrelated to the study; this group therefore had five rather than six ferrets.)

### Ethics statement

This study was carried out in accordance with Animal Welfare Act regulations by the United States Department of Agriculture (USDA) and Public Health Service Policy on Humane Care and Use of Laboratory Animals (PHS Policy) administered by the National Institutes of Health (NIH). All animal research was conducted under a protocol approved by the Centers for Disease Control and Prevention’s Institutional Animal Care and Use Committee (IACUC), in an Association for Assessment and Accreditation of Laboratory Animal Care (AAALAC) International-accredited animal facility. Animal welfare was monitored on a daily basis, and all efforts were made to minimize suffering. Humane endpoints for this study included the presentation of body weight loss exceeding 20% (relative to weight at challenge), indications of neurological symptoms, or a clinical score of 3 in any category based on the system designed by Reuman et al.^53^; however, none of the animals in this study met those criteria.

### Viruses and vaccines

Cell-grown HK/4801 was propagated in the Madin–Darby Canine Kidney (MDCK)—SIAT1 cells,^54^ as described previously.^55^ The virus titer was determined using the 50% tissue culture infectious dose (TCID_50_) assay. Other viruses used in serological assays and in assessment of cell-mediated immunity were propagated in the allantoic cavity of 10-day-old fertile embryonated chicken eggs (Hy-line, Mansfield, GA) at 34 °C for 48 h (72 h for B viruses). Allantoic fluid containing viruses was harvested and frozen at −80 °C until use. Stocks were titered by plaque assays using MDCK cells and expressed as plaque-forming units (pfu). Virus stocks were fully sequenced before use, confirming the absence of mutations other than the expected egg-adaptive changes (N96S, L194P, T160K) in the HA of the egg-grown stocks only; no variants were present in the cell-grown stocks (Supplementary Fig. 1).

Commercial 2016-17 and 2017-18 Northern hemisphere QIV (FLUARIX QIV, GlaxoSmithKline Biologicals, Research Triangle Park, NC: 2016-17 and 2017-18 formula, or FLUZONE QIV, Sanofi Pasteur Inc. Swiftwater, PA: 2016-17 and 2017-18 formula) were used in this study. Both manufacturers’ vaccines contained the same virus strains. The 2016-17 Northern hemisphere QIV included HA and NA from A/California/07/2009 (H1N1)pdm09-like (CA/07), A/Hong Kong/4801/2014 (H3N2)-like (HK/4801); B/Brisbane/60/2008 (B Victoria lineage) (Br/60) and B/Phuket/3073/2013 (B Yamagata lineage) (Ph/3073) viruses.^47^ The commercial 2017-18 Northern hemisphere QIV included HA and NA from A/Michigan/45/2015 (H1N1)pdm09-like instead of CA/07, while other viruses remained the same as in 2016-17.^48^ Appropriate control antigens for serological testing were obtained from the Influenza Reagent Resource (2016-17 and 2017-18 WHO Influenza Reagent Kits, IRR: Influenza Division, WHO Collaborating Center for Surveillance, Epidemiology and Control of Influenza, Centers for Disease Control and Prevention, Atlanta, GA).

### Ferret immunization

Male Fitch ferrets of ~6 months of age, from Triple Farm (Triple F Farms, Sayre, PA) or Marshall BioResources (Marshall BioResources, North Rose, New York), serologically negative by hemagglutination inhibition (HI) assay for currently circulating human influenza H1, H3, and type B viruses, were used in these experiments. Ferrets were arbitrarily assigned to the various groups without formal randomization or blinding. Initial body weights are shown in Supplementary Fig. 2. Immunized ferrets received an adult human dose (0.5 ml, 15 µg of HA) intramuscularly (IM). Control ferrets were mock vaccinated with phosphate-buffered saline (PBS).

### Viral challenge

Baseline weights and temperatures were obtained for the 3 consecutive days prior to challenge and on day 0 (the day of challenge). Body temperatures were measured using an implantable subcutaneous temperature transponder (BioMedic Data Systems, Inc., Seaford, DE). Intranasal inoculation was performed under anesthesia, induced by intramuscular administration of a ketamine–xylazine–atropine mixture, using 2 × 10^5^ TCID_50_ of cell-grown HK/4801, diluted in sterile PBS (1 ml of total volume). Following challenge, ferrets were monitored for changes in body weight and temperature as well as clinical signs of illness on a daily basis for 2 weeks. Blood samples of 200–250 µl were collected from sedated animals on days 0–7, 9, 11, and 13 post challenge. Nasal washes were collected on days 1–7, and 9 post challenge, and infectious viral titers were determined by TCID_50_. Three ferrets from each group were arbitrarily selected for euthanasia on day 2 post challenge, and lungs were fixed in 10% neutral buffered formalin and used for histopathology.

### Serological assays

ELISAs were performed as previously described.^56^ Briefly, ELISA plates were coated overnight with 1 μg/ml of recombinant H1 (A/California/07/2009(H1N1pdm09) (CA/07) (International Reagent Resource) or H3 (HK/4801); eENZYME LLC, Gaithersburg, MD, USA) and blocked with 3% fetal bovine serum (FBS)–PBST. Twofold serial dilutions of 1:100 ferret serum samples were added, followed by horseradish peroxidase-conjugated ferret immunoglobulin (Ig) G (Novus Biologicals, Littleton, CO, USA). O-Phenylenediamine (OPD) solution and H_2_O_2_ were used as the substrate. Absorbance was read at 490 nm, and the last serum dilution that gave a positive/negative optical density readout ratio of >3 was determined as the end-point titer.

Hemagglutination inhibition (HI) and microneutralization (MN) assays were performed as previously described.^55^ Briefly, serum samples were treated with receptor-destroying enzyme (RDE—Denka Seiken Co. Ltd., Tokyo, Japan) and adsorbed with packed turkey red blood cells. HI assays used 0.5% Turkey red blood cells (TRBC) (Lampire Biological Laboratories, Pipersville, PA) against pdmH1 antigens and 0.75% guinea pig red blood cells (gpRBCs) (Lampire Biological Laboratories, Pipersville, PA) against H3 antigens. For H3 antigens, pretreated serum samples were incubated with virus in the presence of 20 nM Oseltamivir carboxylate to eliminate potential interference from NA binding.^57,58^ The HI titer was expressed as the reciprocal of the highest dilution of the serum samples completely inhibiting hemagglutination.

Microneutralization assays (MN) were performed using MDCK-SIAT1 cells.^55^ Briefly, sera were heat inactivated and twofold serial diluted, then mixed with 100 50% tissue culture infection dose (TCID50) of A(H3N2) viruses and incubated at 37 °C 5% CO_2_ for 1 h. The virus–sera mixture was used to infect 1.5 × 10^4^/well Madin–Darby Canine Kidney (MDCK)-SIAT1 cells and incubated for 18–20 h at 37 °C with 5% CO_2_. After cold acetone fixation, the presence of viral protein was quantified by an ELISA using monoclonal antibodies specific to the nucleoproteins (NP) of the influenza A viruses. Antibody titers were calculated as the reciprocal of the highest dilution that neutralized 50% of virus infectivity.

### Cell-mediated immunity

Pre-challenge T-cell responses were evaluated using intracellular cytokine staining (Reber et al.^41^). PBL were stimulated with CA/07 (H1N1pdm09) or egg-grown HK/4801 (H3N2), or with a cocktail of 50 ng/ml phorbol myristate acetate (PMA) and 500 ng/ml ionomycin; for negative controls, we used canine parainfluenza virus, allantoic fluid, and cell media alone. Brefeldin A (Golgi Plug; BD Biosciences, San Diego, CA) was added to cultures for the last 6 h of stimulation. Cells were stained with a live/dead stain (Life Technologies, Grand Island, NY), then with monoclonal antibodies recognizing CD4 (60003-MM02-P, clone: 02, Sino Biological, Beijing, China, 1:20 dilution), CD8 (48-0086-42, clone: OKT-8, eBioscience, San Diego, CA, 1:20 dilution), and IFN-γ (MCA1783A647, clone: CC302, AbD Serotec, Raleigh, NC, 1:10 dilution), and analyzed using a Canto II Flow Cytometer (BD Biosciences). Gating strategy is shown in Supplementary Fig. 6c.

### Histopathology

Respiratory system tissues, including the trachea and lung from euthanized animals, were fixed in 10% neutral buffered formalin and embedded in paraffin. Four-micrometer sections from formalin-fixed, paraffin-embedded specimens were stained with hematoxylin and eosin (H&E) for histopathologic evaluation. Each lung sample was given a score based on degrees of inflammation. (0 =;no inflammation; 1 = mild inflammation; 2 = moderate inflammation).

### Peripheral blood leukocyte analysis

Blood samples of 200–250 µl were collected in EDTA Vacutainer tubes (Tyco HealthCare Group LP, Mansfield, MA) from sedated ferrets on days 0–7, 9, 11, and 13 post challenge. Peripheral blood leukocyte (PBL) purification and flow cytometry were performed (Music et al.^40^, Music et al.^39^), using monoclonal antibodies recognizing ferret CD4 (Sino Biological Inc., Beijing, China), or cross-reacting with ferret CD8 (eBioscience, San Diego, CA) or CD11b (clone M1/70, eBioscience, 1:20 dilution). Gating strategies are shown in Supplementary Fig. 6a, b.

### Limitations

Interpretation of these experiments may be limited by the lack of formal randomization and blinding, the use of influenza-naive ferrets which may not reflect exposure of humans to multiple influenza strains over many years, and the specific context of antigenic relatedness of the particular vaccines and challenge virus used, which in humans may be different in each influenza season.

### Statistical analyses

Statistical analysis was performed using a linear mixed model with repeated measures implemented either with SAS (version 9.4) or the lmerTest package (version 3) in R (version 3.5). For comparison of HI and MN titers against egg- versus cell-grown virus, a two-sided Student's *t* test was used.

### Reporting summary

Further information on experimental design is available in the Nature Research Reporting Summary linked to this article.

## Supplementary information

Supplementary Figures

Reporting Summary

## Data Availability

The data sets generated and analyzed during this study are available from the corresponding author on reasonable request.

## Supplementary information

**Supplementary information** accompanies the paper on the *npj Vaccines* website (10.1038/s41541-019-0123-7).
