# Supplementary material for: Repeated vaccination against matched H3N2 influenza virus gives less protection than single vaccination in ferrets
Source: NPJ Vaccines. 2019 Jul 9;4:28. doi: 10.1038/s41541-019-0123-7 (PMC6616337; doi:10.1038/s41541-019-0123-7)
Supplement: Supplementary file 1 — Supplementary Figures [file 41541_2019_123_MOESM1_ESM.pdf]

Supplementary Figure 1

Sequences of cell- and egg-grown A/Hong Kong/4801/2014(H3N2)

	1	2	3	4	5	6	7	8	9	10	11	12	13	14	15	16	17	18	19	20	21	22	23	24	25	26	27	28	29	30	31	32	33	34	35	36	37	38	39	40	41	42	43	44	45	46	47	48	49	50	51	52	53	54	55	56	57	58	59	60
Cell grown	M	K	T	I	I	A	L	S	Y	I	L	C	L	V	F	A	Q	K	I	P	G	N	D	N	S	T	A	T	L	C	L	G	H	H	A	V	P	N	G	T	I	V	K	T	I	T	N	D	R	I	E	V	T	N	A	T	E	L	V	Q
Egg grown	M	K	T	I	I	A	L	S	Y	I	L	C	L	V	F	A	Q	K	I	P	G	N	D	N	S	T	A	T	L	C	L	G	H	H	A	V	P	N	G	T	I	V	K	T	I	T	N	D	R	I	E	V	T	N	A	T	E	L	V	Q

	61	62	63	64	65	66	67	68	69	70	71	72	73	74	75	76	77	78	79	80	81	82	83	84	85	86	87	88	89	90	91	92	93	94	95	96	97	98	99	100	101	102	103	104	105	106	107	108	109	110	111	112	113	114	115	116	117	118	119	120
Cell grown	N	S	S	I	G	E	I	C	D	S	P	H	Q	I	L	D	G	E	N	C	T	L	I	D	A	L	L	G	D	P	Q	C	D	G	F	Q	N	K	K	W	D	L	F	V	E	R	S	K	A	Y	S	N	C	Y	P	Y	D	V	P	D
Egg grown	N	S	S	I	G	E	I	C	D	S	P	H	Q	I	L	D	G	E	N	C	T	L	I	D	A	L	L	G	D	P	Q	C	D	G	F	Q	N	K	K	W	D	L	F	V	E	R	S	K	A	Y	S	S	C	Y	P	Y	D	V	P	D

	121	122	123	124	125	126	127	128	129	130	131	132	133	134	135	136	137	138	139	140	141	142	143	144	145	146	147	148	149	150	151	152	153	154	155	156	157	158	159	160	161	162	163	164	165	166	167	168	169	170	171	172	173	174	175	176	177	178	179	180
Cell grown	Y	A	S	L	R	S	L	V	A	S	S	G	T	L	E	F	N	N	E	S	F	N	W	T	G	V	T	Q	N	G	T	S	S	A	C	I	R	R	S	S	S	S	F	F	S	R	L	N	W	L	T	H	L	N	Y	T	Y	P	A	L
Egg grown	Y	A	S	L	R	S	L	V	A	S	S	G	T	L	E	F	N	N	E	S	F	N	W	T	G	V	T	Q	N	G	T	S	S	A	C	I	R	R	S	S	S	S	F	F	S	R	L	N	W	L	T	H	L	N	Y	K	Y	P	A	L

	181	182	183	184	185	186	187	188	189	190	191	192	193	194	195	196	197	198	199	200	201	202	203	204	205	206	207	208	209	210	211	212	213	214	215	216	217	218	219	220	221	222	223	224	225	226	227	228	229	230	231	232	233	234	235	236	237	238	239	240
Cell grown	N	V	T	M	P	N	N	E	Q	F	D	K	L	Y	I	W	G	V	H	H	P	G	T	D	K	D	Q	I	F	L	Y	A	Q	S	S	G	R	I	T	V	S	T	K	R	S	Q	Q	A	V	I	P	N	I	G	S	R	P	R	I	R
Egg grown	N	V	T	M	P	N	N	E	Q	F	D	K	L	Y	I	W	G	V	H	H	P	G	T	D	K	D	Q	I	F	P	Y	A	Q	S	S	G	R	I	T	V	S	T	K	R	S	Q	Q	A	V	I	P	N	I	G	S	R	P	R	I	R

	241	242	243	244	245	246	247	248	249	250	251	252	253	254	255	256	257	258	259	260	261	262	263	264	265	266	267	268	269	270	271	272	273	274	275	276	277	278	279	280	281	282	283	284	285	286	287	288	289	290	291	292	293	294	295	296	297	298	299	300
Cell grown	D	I	P	S	R	I	S	I	Y	W	T	I	V	K	P	G	D	I	L	L	I	N	S	T	G	N	L	I	A	P	R	G	Y	F	K	I	R	S	G	K	S	S	I	M	R	S	D	A	P	I	G	K	C	K	S	E	C	I	T	P
Egg grown	D	I	P	S	R	I	S	I	Y	W	T	I	V	K	P	G	D	I	L	L	I	N	S	T	G	N	L	I	A	P	R	G	Y	F	K	I	R	S	G	K	S	S	I	M	R	S	D	A	P	I	G	K	C	K	S	E	C	I	T	P

	301	302	303	304	305	306	307	308	309	310	311	312	313	314	315	316	317	318	319	320	321	322	323	324	325	326	327	328	329	330	331	332	333	334	335	336	337	338	339	340	341	342	343	344	345	346	347	348	349	350	351	352	353	354	355	356	357	358	359	360
Cell grown	N	G	S	I	P	N	D	K	P	F	Q	N	V	N	R	I	T	Y	G	A	C	P	R	Y	V	K	H	S	T	L	K	L	A	T	G	M	R	N	V	P	E	K	Q	T	R	G	I	F	G	A	I	A	G	F	I	E	N	G	W	E
Egg grown	N	G	S	I	P	N	D	K	P	F	Q	N	V	N	R	I	T	Y	G	A	C	P	R	Y	V	K	H	S	T	L	K	L	A	T	G	M	R	N	V	P	E	K	Q	T	R	G	I	F	G	A	I	A	G	F	I	E	N	G	W	E

	361	362	363	364	365	366	367	368	369	370	371	372	373	374	375	376	377	378	379	380	381	382	383	384	385	386	387	388	389	390	391	392	393	394	395	396	397	398	399	400	401	402	403	404	405	406	407	408	409	410	411	412	413	414	415	416	417	418	419	420
Cell grown	G	M	V	D	G	W	Y	G	F	R	H	Q	N	S	E	G	R	G	Q	A	A	D	L	K	S	T	Q	A	A	I	D	Q	I	N	G	K	L	N	R	L	I	G	K	T	N	E	K	F	H	Q	I	E	K	E	F	S	E	V	E	G
Egg grown	G	M	V	D	G	W	Y	G	F	R	H	Q	N	S	E	G	R	G	Q	A	A	D	L	K	S	T	Q	A	A	I	D	Q	I	N	G	K	L	N	R	L	I	G	K	T	N	E	K	F	H	Q	I	E	K	E	F	S	E	V	E	G

	421	422	423	424	425	426	427	428	429	430	431	432	433	434	435	436	437	438	439	440	441	442	443	444	445	446	447	448	449	450	451	452	453	454	455	456	457	458	459	460	461	462	463	464	465	466	467	468	469	470	471	472	473	474	475	476	477	478	479	480
Cell grown	R	I	Q	D	L	E	K	Y	V	E	D	T	K	I	D	L	W	S	Y	N	A	E	L	L	V	A	L	E	N	Q	H	T	I	D	L	T	D	S	E	M	N	K	L	F	E	K	T	K	K	Q	L	R	E	N	A	E	D	M	G	N
Egg grown	R	I	Q	D	L	E	K	Y	V	E	D	T	K	I	D	L	W	S	Y	N	A	E	L	L	V	A	L	E	N	Q	H	T	I	D	L	T	D	S	E	M	N	K	L	F	E	K	T	K	K	Q	L	R	E	N	A	E	D	M	G	N

	481	482	483	484	485	486	487	488	489	490	491	492	493	494	495	496	497	498	499	500	501	502	503	504	505	506	507	508	509	510	511	512	513	514	515	516	517	518	519	520	521	522	523	524	525	526	527	528	529	530	531	532	533	534	535	536	537	538	539	540
Cell grown	G	C	F	K	I	Y	H	K	C	D	N	A	C	I	G	S	I	R	N	G	T	Y	D	H	N	V	Y	R	D	E	A	L	N	N	R	F	Q	I	K	G	V	E	L	K	S	G	Y	K	D	W	I	L	W	I	S	F	A	I	S	C
Egg grown	G	C	F	K	I	Y	H	K	C	D	N	A	C	I	G	S	I	R	N	G	T	Y	D	H	N	V	Y	R	D	E	A	L	N	N	R	F	Q	I	K	G	V	E	L	K	S	G	Y	K	D	W	I	L	W	I	S	F	A	I	S	C

	541	542	543	544	545	546	547	548	549	550	551	552	553	554	555	556	557	558	559	560	561	562	563	564	565	566
Cell grown	F	L	L	C	V	A	L	L	G	F	I	M	W	A	C	Q	K	G	N	I	R	C	N	I	C	I
Egg grown	F	L	L	C	V	A	L	L	G	F	I	M	W	A	C	Q	K	G	N	I	R	C	N	I	C	I

Supplementary Figure 1. Sequence of hemagglutinin from egg- and cell-grown A/Hong Kong/4801/2014(H3N2). Virus stocks used in these experiments were sequenced to confirm the absence of unexpected mutations. The expected egg-adaptive mutations N96S, L194P, and T160K are highlighted.

Supplementary Figure 2

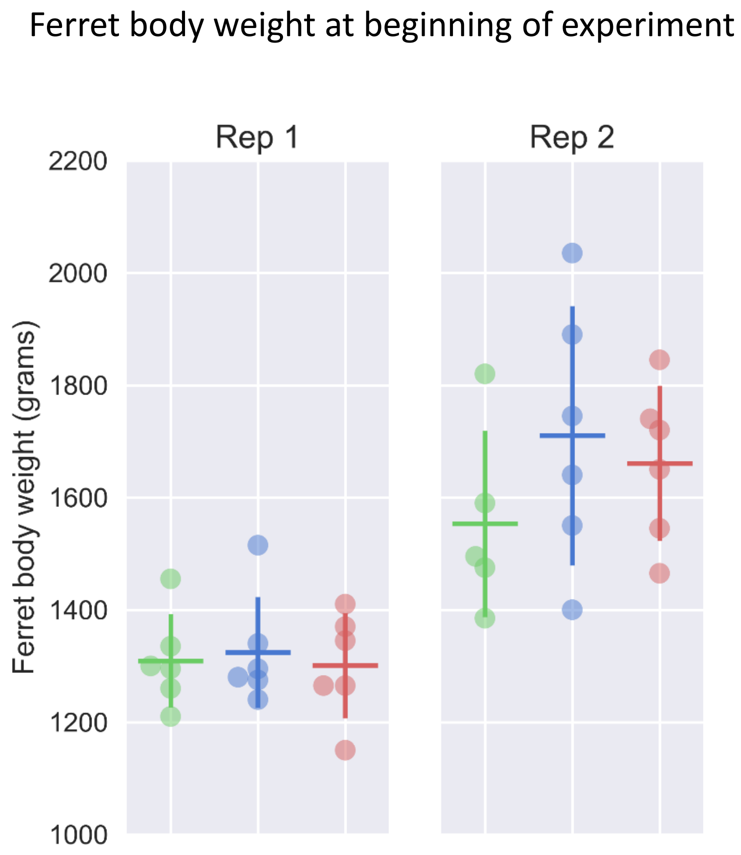

Supplementary Figure 2. Ferret body weights at the onset of the experiments. Each dot represents one ferret; horizontal bars represent mean values; error bars represent one standard deviation.

Supplementary Figure 3

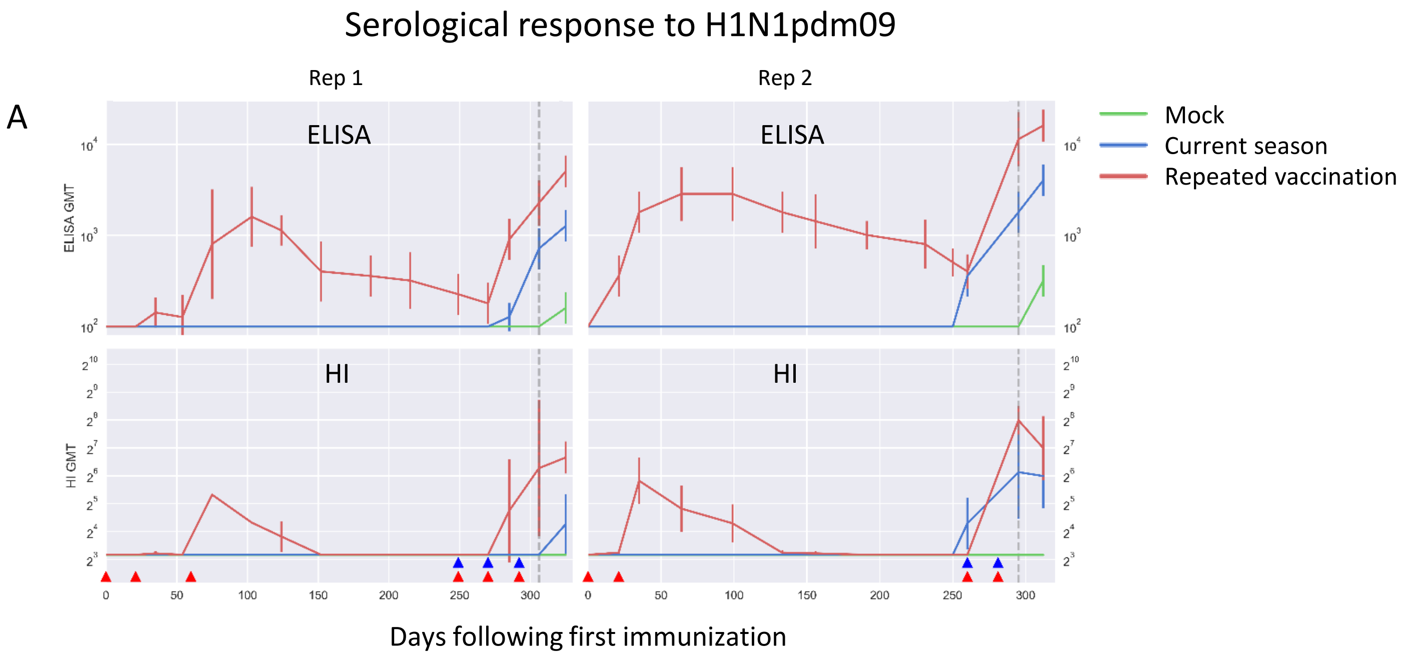

Supplementary Figure 3. Serological responses to (H1N1)pdm09. Ferrets were vaccinated with commercial quadrivalent inactivated vaccine (QIV) containing (Rep 1) A/California/07/2009 (H1N1)pdm09 or (Rep 2) the antigenically similar A/Michigan/45/2015 (H1N1)pdm09 as well as HK/4801. Geometric mean titers against CA/07 measured by ELISA (top panels) or HI (bottom panels) are shown

Supplementary Figure 4

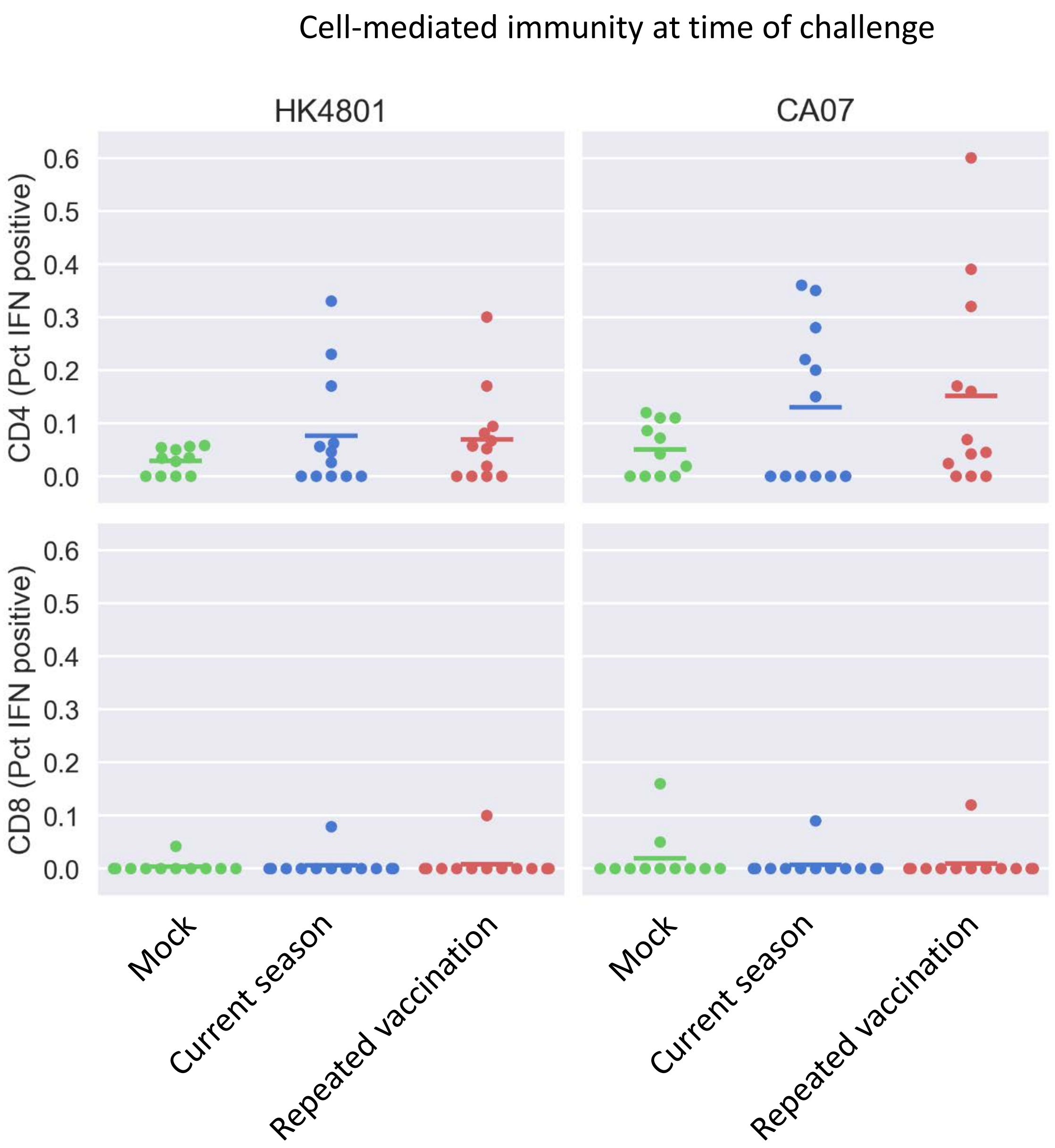

Supplementary Figure 4. Cell mediated immunity before influenza challenge. Following immunization with QIV by the various regimens as described, peripheral blood leukocytes were collected and the percent of CD3/CD4+ (top row) or CD3/CD8+ (bottom row) T cells expressing interferon-γ after overnight stimulation with HK/4801(H3N2) (left panels) or influenza A/California/07/2009(H1N1pdm09) (right panels) was measured by flow cytometry. The difference between groups was not statistically significant ($p > 0.05$). Each dot represents an individual ferret; the bar represents the mean for each group.

Supplementary Figure 5

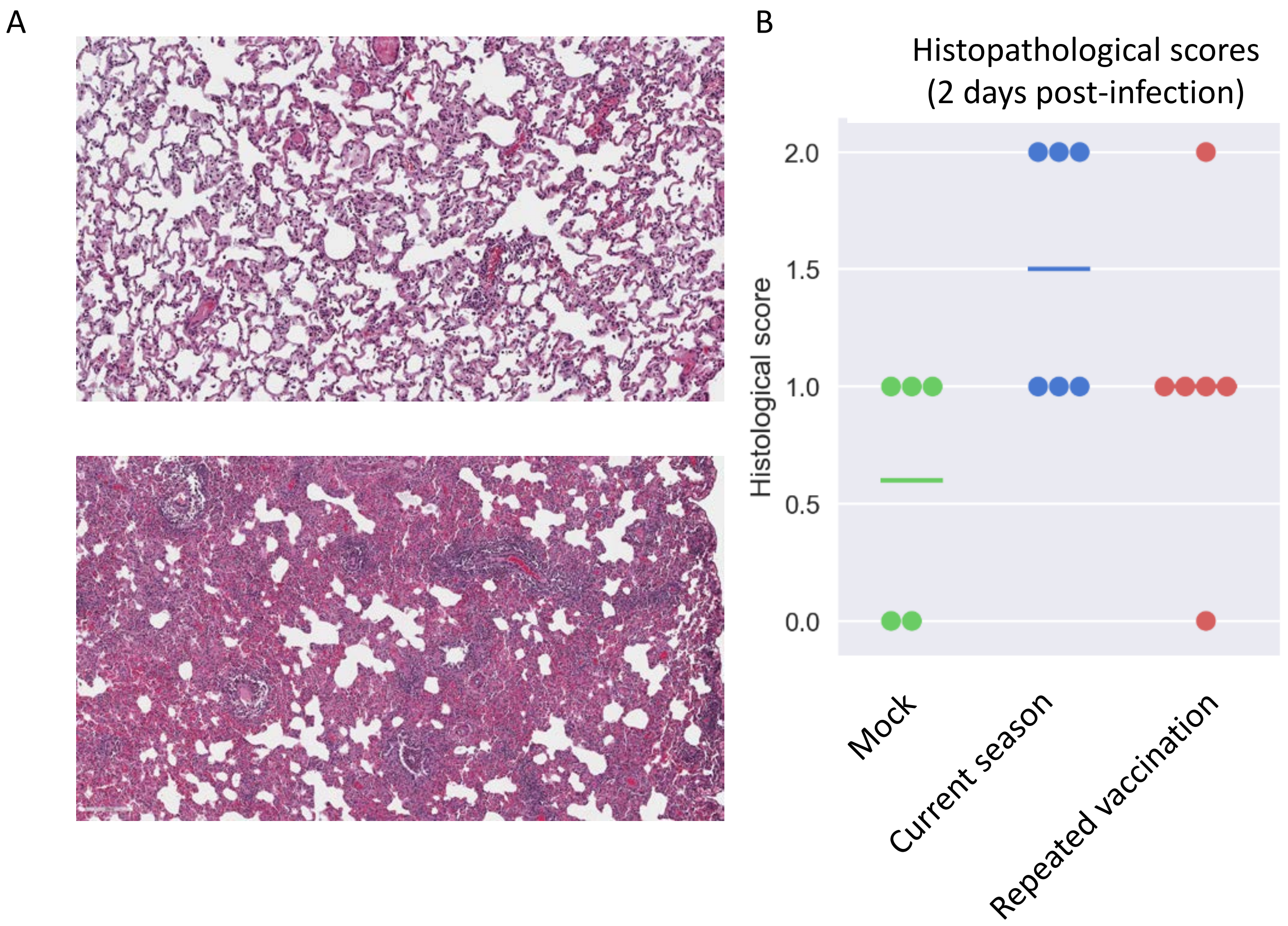

Supplementary Figure 5. Histopathologic evaluation of ferret tissues. Two days after intranasal challenge with cell-grown HK/4801, three ferrets per group were sacrificed and lungs were collected and processed for histopathology. **(A)** Representative tissue samples to illustrate the range of inflammation seen. The upper panel shows focal mild perivascular infiltrates. The lower panel shows moderate interstitial and perivacular infiltrates. (Hematoxylin and Eosin stain, Scale Bars: 100 mm) **(B)** Each lung sample was given a score based on degrees of inflammation (0 = no inflammation; 1 = mild inflammation; 2 = moderate inflammation). Each dot represents an individual ferret; the bar represents the mean for each group. The difference between groups was not statistically significant ($p > 0.05$). Each dot represents an individual ferret; the bar represents the mean for each group.

Supplementary Figure 6

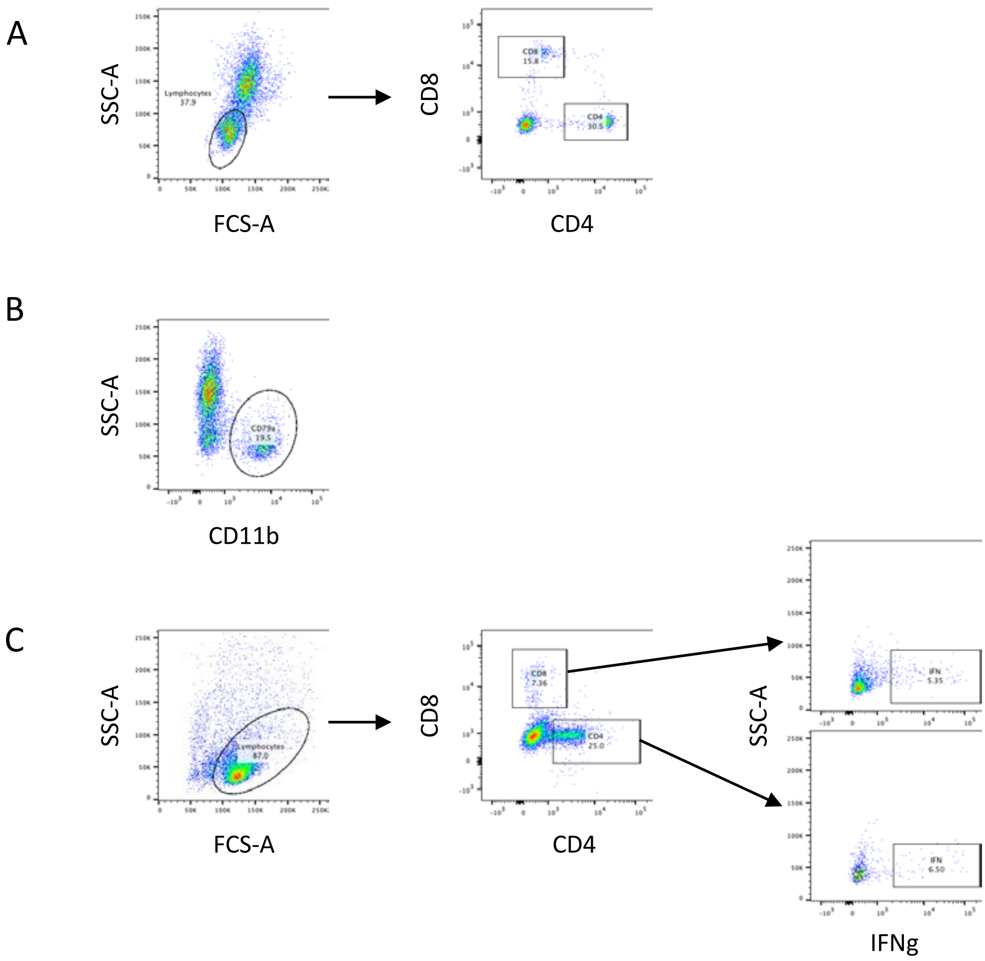

Supplementary Figure 6. Gating strategies for flow cytometry. (A) For peripheral blood lymphocyte counts, lymphocytes were selected based on forward and side scatter, and T lymphocytes were stained with anti-CD4 and anti-CD8. **(B)** For peripheral blood granulocyte counts, granulocytes were stained with CD11b. **(C)** For intracellular cytokines, following stimulation and permeablization, lymphocytes were selected based on forward and side scatter, T lymphocytes were gated using anti-CD4 and anti-CD8, and stimulated cells were identified with intracellular interferon γ staining.
